# Comparative Analysis of Multi-Organ Failure Trajectories Following Heart Transplantation and HeartMate 3 Implantation: A 1-Year Postprocedural Follow-Up Study Utilizing the MELD-XI Scale

**DOI:** 10.3390/jcm14175933

**Published:** 2025-08-22

**Authors:** Mateusz Sokolski, Jakub Ptak, Małgorzata Makieła, Maciej Szwajkowski, Mateusz Waloszczyk, Kacper Wiśniewski, Joanna Gontarczyk, Paulina Makowska, Dominik Krupka, Natalia Sitko, Magdalena Cielecka, Mateusz Rakowski, Maciej Bochenek, Roman Przybylski, Michał Zakliczyński

**Affiliations:** 1 Institute of Heart Diseases, Jan Mikulicz Radecki University Hospital Wroclaw, 50556 Wroclaw, Polandmichal.zakliczynski@umw.edu.pl (M.Z.); 2 Clinical Department of Heart Transplantation and Mechanical Circulatory Support, Department of Cardiac, Surgery and Heart Transplantation, Institute of Heart Diseases, Faculty of Medicine, Wroclaw Medical University, 50368 Wroclaw, Poland; 3 Institute of Heart Diseases, Student Scientific Club of Transplantology and Advanced Therapies of Heart Failure, Faculty of Medicine, Wroclaw Medical University, 50368 Wroclaw, Poland; 4 Faculty of Health Sciences, Wroclaw Medical University, 50368 Wroclaw, Poland

**Keywords:** MELD-XI, HTx, LVAD, multi-organ failure

## Abstract

**Background**: Multi-organ failure (MOF) is a common complication of advanced heart failure (HF), significantly influencing patient prognosis. This study aimed to assess and compare the impact of orthotopic heart transplantation (HTx) and left ventricular assist device (LVAD) implantation on the severity of MOF, as measured by the model for end-stage liver disease excluding INR (MELD-XI) score. **Methods**: Data from 1 month before to 1 year after HTx or LVAD implantation were analysed. The MELD-XI score was calculated using average bilirubin and creatinine values. Comparative assessments of MELD-XI scores were performed within the HTx and LVAD groups at various time points pre- and post-procedure. **Results**: The analysis included 107 HTx patients and 30 LVAD patients. The median MELD-XI score 1 month pre-procedure was 11.7 (9.4–16.7) in all patients. There were no significant differences in MELD-XI scores between the groups at 3-, 6-, and 12-month follow-ups. However, a significant difference was observed 1 month post-procedure [HTx: 14.8 (9.4–17.7) vs. LVAD: 11.2 (7.3–14.9), *p* = 0.02]. In the LVAD group, a significant decrease in MELD-XI score was noted for 3 months post-procedure compared to 1 month pre-procedure (*p* < 0.001), whereas at 6- and 12-month follow-ups the score did not differ from pre-procedural scores. In the HTx group, significant decreases in MELD-XI scores were observed from 3 months, 6 months, and 1 year post-procedure compared to 1 month pre-procedure (*p* < 0.002). **Conclusions**: The MELD-XI scale reveals different MOF trajectories between HTx and LVAD recipients. Both interventions lead to early improvements in liver and kidney function, with sustained benefits in HTx patients, highlighting the distinct impacts on organ function.

## 1. Introduction

Heart failure (HF), particularly in advanced stages, contributes to a cascade of multi-organ failure (MOF), exerting a profound impact on disease progression and adversely influencing outcomes [1,2]. The intricate interplay between HF and the liver and kidneys gives rise to cardiohepatic and cardiorenal syndromes, further complicating the clinical landscape [3].

The Model of End-Stage Liver Disease (MELD) was originally designed for prognostic assessment in advanced liver disease [4,5]. Over time, the MELD scale has undergone iterative updates and refinements. The MELD scoring system enables simultaneous assessment of both hepatic and renal function, offering a valuable tool for predicting outcomes in patients with HF. As a result, indicators of liver impairment and the MELD score have become key elements in evaluating risk among individuals with HF [6,7,8]. The MELD XI is a scale that excludes the international normalized ratio (INR). It integrates parameters of liver and kidney function, which allows for the assessment of the severity of organ dysfunction, taking into account both of these important systems. Its utility has been demonstrated in various clinical settings. For instance, Amdani et al. found that both pre- and post-implant MELD-XI scores were significantly associated with survival in pediatric patients undergoing ventricular assist device implantation, highlighting the broader applicability of the scale [9]. Additionally, it has been shown that the MELD score can serve as an indicator of MOF [10]. The mechanism of MOF in advanced HF involves complex interactions between cardiac dysfunction and other organ systems. Elevated venous pressure, a hallmark of HF, negatively impacts both renal and hepatic function. In the kidneys, increased venous pressure leads to reduced glomerular filtration due to elevated pressures transmitted to the efferent arterioles, resulting in decreased filtration pressure and kidney damage [11]. In the liver, increased venous pressure causes hepatocyte atrophy and perisinusoidal edema, impairing the diffusion of oxygen and nutrients to hepatocytes [12]. Various biochemical markers have been explored in this context. A recent study by Qing et al. identified the direct-to-total bilirubin ratio as a predictor of right heart failure following LVAD implantation, underlining the prognostic relevance of liver function parameters in patients receiving mechanical circulatory support [13]. Previous versions of the MELD have demonstrated its prognostic value, especially in the context of patients undergoing heart transplantation (HTx) [8,10,14]. However, the independent prognostic role of MELD-XI in post-HTx and left ventricular assist device (LVAD) treatment remains underexplored. This study aims to elucidate the trajectory of MOF in individuals undergoing HTx. The gold standard of advanced HF or LVAD—HeartMate 3 implantation—is currently the best alternative to HTx, utilizing the MELD-XI scale over a 1-year follow-up period.

## 2. Materials and Methods

### 2.1. Study Design

The study was conducted in four structured stages. First, key laboratory parameters were retrospectively collected and entered into a database. Mean values for these parameters were subsequently calculated, covering the period from 1 month prior to HTx or LVAD implantation to twelve months following hospital discharge. In the third step, MELD-XI scores were computed using the averaged data. Finally, the complete dataset was subjected to detailed statistical analysis.

### 2.2. Participants and Setting

The cohort consisted of patients treated with either HTx or LVAD implantation between 25 February 2021 and 15 June 2023, amounting to a total of 137 individuals (107-HTx; 30-LVAD), all of whom completed 6-month follow-up. Complete 1-year follow-up was achieved by 122 patients (96-HTx; 26-LVAD). In 12 months, 28 (23%) {22 (23%) HTx; six (23%) LVAD} patients died, one (1%) had re-HTx, and eight (31%) LVAD patients had HTx.

Patients with incomplete data required for MELD-XI score calculation were to be excluded; however, all individuals had complete datasets, and no exclusions were necessary. All patients received routine clinical evaluation and guideline-directed therapy during their hospitalization. Follow-up data at 3, 6, and 12 months were obtained during scheduled hospital visits in accordance with the recommendations of the International Society for Heart and Lung Transplantation [15]. The reason for using the MELD-XI scale instead of the new MELD-3.0 scale is that the previous scale does not include the INR. In the pre-transplant period, 13 patients with heart failure had clinical indications for anticoagulation therapy using either novel oral anticoagulants or vitamin K antagonists (VKA). All LVAD patients after the implantation had these indications.

The MELD-XI score was calculated using the formula:

MELD-XI = 5.11 × ln (serum bilirubin in mg/dL) + 11.76 × ln (serum creatinine in mg/dL) + 9.44

The MELD 3.0 score was also calculated for HTx group using the formula:

1.33 (if woman) + 4.56 × ln[bilirubin] + 0.82 × (137 − [Na]) − 0.24 × (137 − [Na]) × ln[bilirubin] + 11.14 × ln[creatinine] + 9.09 × ln(INR) + 1.85 × (3.5 − [albumin]) − 1.83 × (3.5 − [albumin]) × ln[creatinine] + 6.

### 2.3. Statistical Methods

Continuous variables with normal distribution were expressed as means ± standard deviation. For variables exhibiting a skewed distribution, medians along with lower and upper quartiles were employed. The Shapiro–Wilk test was used to assess the normality of distributions. For between-group comparisons, the Mann–Whitney U test was employed for variables with a skewed distribution, considering the non-parametric nature of the latter, while the T-test was applied for normally distributed variables. Categorical variables were summarized as counts and percentages, and group differences were evaluated using the chi-square test. For variables with small expected events (<5), the Fisher exact test was utilized. To evaluate changes in repeated measurements of non-normally distributed variables over time, the Friedman test was applied.

**Figure 1:**
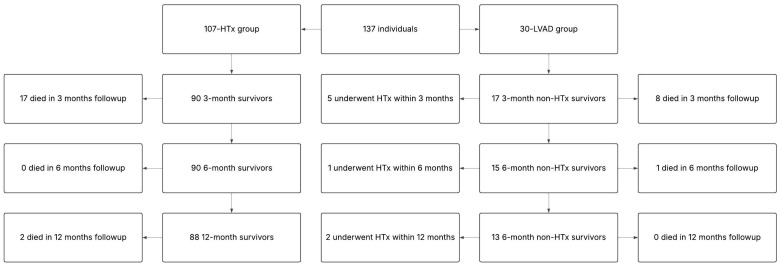
Flow chart.

A significance threshold of *p* < 0.05 was considered statistically significant. All statistical analyses were performed using the STATISTICA (version 14.0.0.15) data analysis software system (StatSoft, Inc., Tulsa, OK, USA) (Figure 1).

## 3. Results

### 3.1. Descriptive Data

The majority of the cohort was men (85%), median age of 54 (47–62) years, of whom 63 (46%) were diagnosed with hypertension, 48 (35%) with chronic kidney disease (CKD), and 37 (27%) with diabetes. The most common HF etiology was ischemic (48%). The median N-terminal pro B-type natriuretic peptide (NTproBNP) was 4665.0 (2270.2–8975.6) pg/mL, bilirubin 1.25 (0.82–2.05) mg/dL, creatinine 1.15 (0.97–1.42) mg/dL, Hb 12.73 (10.92–14.23) g/dL, INR 1.29 (1.11–1.67), C-reactive protein (CRP) 13.4 (3.82–48.22) mg/L, albumin 3.5 (3.0–3.95) g/dL, sodium level (Na) 136.33 (133.71–139.67) mmol/L, and troponin 36.2 (15.86–958.92) ng/L. The median MELD-XI before the HTx was 11.68 (9.21–16.74), and before the LVAD was 12.976 (9.63–16.02).

Mean central venous pressure (CVP) was 10.71 (±6.18) mmHg, median cardiac output (CO) measured with the thermodilution technique 4.07 L/min (3.47–4.70), mean pulmonary artery pressure (MPAP) 30.25 (±10.11) mmHg, and pulmonary vascular resistance (PVR) 2.27 (1.81–3.15) WU. All of the described data, with an indication of the numbers of participants with missing data for each variable of interest, are presented [Table 1, Table 2 and Table 3].

An analysis of pharmacotherapy before and after HTx and LVAD procedures revealed significant changes in the use of many key medications, as shown in Table 4 for the HTx group and in Table 5 for the LVAD group. Additionally, LVAD patients continued to require HF treatment according to guidelines and anticoagulation therapy, whereas HTx patients did not.

### 3.2. Main Results

The median MELD-XI score 1 month pre-procedure was 11.7 (9.4–16.7) in both groups and 11.7 (9.2–16.7) in the HTx group, comparable to 13.0 (9.6–16.1) in the LVAD group (*p* = 0.53). There were no significant differences in MELD-XI scores between the groups at 3-, 6-, and 12-month follow-ups [HTx vs. LVAD respectively: 9.3 (6.7–13.3) vs. 9.4 (6.4–13.6), *p* = 0.95; 9.4 (6.7–12.5) vs. 12.0 (6.7–17.0), *p* = 0.23; 10.4 (7.2–12.9) vs. 12.2 (6.5–14.1), *p* = 0.76]. However, a significant difference was observed 1 month post-procedure [HTx: 14.8 (9.4–17.7) vs. LVAD: 11.2 (7.3–14.9), *p* = 0.02] [Table 6].

**Figure 2:**
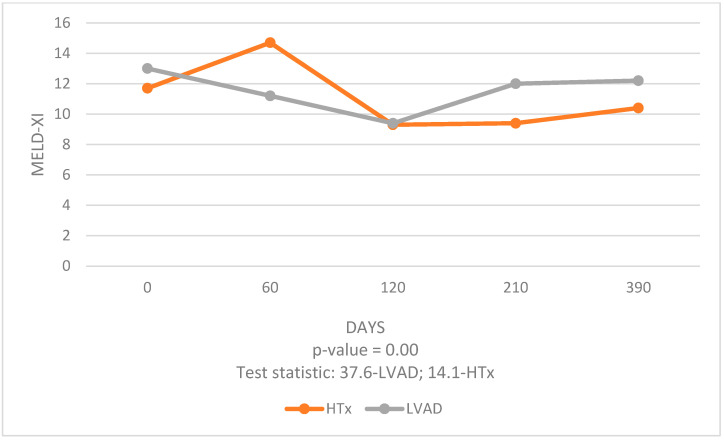
MELD-XI Trajectory.

In the LVAD group, a significant decrease in MELD-XI score was noted at 3 months post-procedure compared to 1 month pre-procedure (*p* < 0.001), whereas at 6- and 12-month follow-ups the score did not differ statistically from pre-procedural scores. [Figure 2]. In the HTx group, significant decreases in MELD-XI scores were observed at 3 months, 6 months, and 1 year post-procedure compared to 1 month pre-procedure (*p* < 0.002 in all comparisons) [Table 7].

### 3.3. Liver and Kidney Function

Markers of liver and kidneys damage or function were compared at 3-, 6- and 12-month follow-up with the value from 1 month before the intervention. The values of bilirubin, alanine aminotransferase (ALT), aspartate aminotransferase (AST), gamma-glutamylotranspeptydase (GGTP), creatinine and urea were analysed. No kidney parameters improved over the whole observation period in both groups. The improvement was observed only in ALT and bilirubin at 3-month follow-up in the LVAD group [Table 8 and Table 9], while in the HTx group ALT, AST, GGTP, and Bil improved at all of the timepoints [Table 10 and Table 11].

### 3.4. Complications

Early complications and rehospitalization rates, as presented in Table 12, slightly favored HTx over LVAD implantation. Half of deaths were attributed to right or left heart failure, while 25% were due to sepsis or infectious complications.

## 4. Discussion

The analysis of data over a 1-year period post-procedure allowed us to compare the effects of these two different interventions on liver and kidney function. Our study revealed significant findings regarding the temporal changes in MELD-XI scores post-procedure. Both HTx and LVAD groups exhibited a significant early improvement in MELD-XI scores, indicative of enhanced liver and kidney function. However, the trajectory of these improvements differed between the two groups. LVAD patients experienced a significant decrease in MELD-XI scores at 3 months post-procedure compared to 1 month pre-procedure, suggesting an initial benefit. However, this improvement did not persist at 6 and 12 months, indicating less durable organ function stabilization. In contrast, HTx patients showed sustained improvements in MELD-XI scores at 3, 6, and 12 months post-procedure compared to 1 month pre-procedure. This consistent decline in MELD-XI scores highlights the long-term benefits of HTx in stabilizing and improving liver and kidney function.

The early improvement in the LVAD group can be attributed to the immediate hemodynamic stabilization provided by the device, which mechanically unloads the heart and enhances CO. However, the durability of these improvements may be compromised by complications associated with long-term LVAD use, such as infections, bleeding, and thromboembolic events, which can adversely affect organ function [16,17,18,19,20].

HTx, despite its associated risks of early postoperative complications such as graft rejection and infections, offers more stable long-term benefits. The use of immunosuppressive therapy and close patient monitoring post-HTx contribute to the sustained improvement in organ function, as evidenced by the continuous decline in MELD-XI scores [21]. It is commonly known that calcineurin inhibitors are known to induce nephrotoxicity. The immunosuppressive regimen administered to HTx recipients in our cohort followed a standard triple-therapy protocol. This included a calcineurin inhibitor—most commonly tacrolimus (98%), with target trough blood levels of 10–15 ng/mL, and less frequently cyclosporine (2%), with target levels of 200–300 ng/mL. In addition, patients received an antiproliferative agent, typically mycophenolate mofetil (94%), and glucocorticosteroids (100%), most often prednisone, which was tapered gradually and continued at reduced doses for up to 1 year post-transplant. Dosing adjustments for all immunosuppressants were individualized based on serum drug concentration monitoring during protocolized follow-up visits.

HTx remains the cornerstone treatment for selected patients with end-stage HF [22]. Given the increasing number of patients with advanced HF and the stagnation in the availability of donor hearts, HTx is viable for a limited number of patients. Advanced heart assist devices like LVADs are emerging as a viable alternative, offering patients with advanced HF a chance for improved survival [23,24]. However, our study showed less lasting improvement in liver and kidney function in LVAD patients and higher early complications rates resulting in rehospitalizations compared with HTx patients. Nevertheless, we did not demonstrate statistically significant differences in MELD-XI scores between the LVAD and HTx groups in the long term.

It was also observed that liver functions improved more significantly than kidney functions, particularly in the HTx group. This distinction suggests that HTx has a more pronounced positive impact on hepatic function, possibly due to the liver’s greater ability to recover from the hemodynamic changes and venous congestion associated with advanced HF.

It is important to note that our study has some limitations—the number of patients in the LVAD group was smaller than in the HTx group, which may affect the overall statistical power of our analyses. Despite these limitations, our study provides valuable insights into the MOF trajectory post-HTx and LVAD implantation. Comparisons between the HTx and LVAD groups revealed no significant differences, except for body mass index (BMI), body mass, sex, CKD and NTproBNP levels. Differences in BMI and gender between the HTx and LVAD groups may be due to the difficulty in finding a suitable heart donor for patients with higher body weight and larger sizes. For men, who statistically have higher body mass and BMIs, there is a greater likelihood of being classified for LVAD therapy rather than HTx. This may explain the higher number of men and the higher BMI in the LVAD patient group compared to the HTx group. Although a higher percentage of patients with CKD was recorded in the LVAD group compared to the HTx group, it should be noted that glomerular filtration rate (GFR) rates and creatinine levels did not differ significantly between the groups. This suggests that the difference in the prevalence of CKD may be due to differences in medical documentation rather than an actual difference in kidney function between the groups. The LVAD group exhibited higher NTproBNP concentrations than the HTx group; however, both groups had values significantly above normal, highlighting the severity of the disease in the entire study cohort.

At the time of data collection for our study, LVAD implantation was not indicated as a destination therapy in our centre. Therefore, patients who received an LVAD were those who did not have suitable heart donors available and were considered for bridge-to-transplant therapy. Selection for LVAD was primarily driven by clinical urgency and the lack of a compatible donor, which was often due to factors such as rarer blood type or higher body mass index (BMI), making it more difficult to match with a donor organ. Consequently, while both groups had advanced heart failure, the HTx group consisted of patients for whom a suitable donor was available in a timely manner.

The interpretation of MELD-XI scores should consider the influence of age, comorbidities, and HF severity, as these factors may independently affect liver and kidney function. Their interplay can modulate MELD-XI trajectories over time and partially explain individual variability in response to both HTx and LVAD therapy.

## 5. Conclusions

This study shows that utilizing the MELD-XI scale reveals different MOF trajectories between HTx and LVAD recipients. Both treatments resulted in improved liver and kidney function. In the LVAD group, the improvement was shorter than in the HTx group, but in the long term, no significant differences in MELD-XI scores were observed between the LVAD and HTx groups. It was also observed that liver functions improved more than kidney functions, especially in the HTx group.

## Figures and Tables

**Table 1:** Characteristics of the study population.

Parameter	All Patients (*n* = 137)	Valid *n*	HTx (*n* = 107)	LVAD (*n* = 30)	*p*-Value
Age, years (IQR)	54 (47–62)	137	53 (44–63)	56 (49–62)	0.33
BMI, kg/m^2^ (SD)	26.8 (4.3)	127	26.1 (4.0)	29.5 (4.4)	0.00
Body mass, kg (SD)	81.2 (16.0)	132	78.1 (15.2)	92.1 (14.0)	0.00
Ischemic HF etiology, n (%)	66 (48)	137	47 (44)	19 (63.3)	0.06
Sex, male (%)	116 (85)	137	87 (81.3)	29 (96.7)	0.04
Hypertension, n (%)	63 (46)	137	47 (44)	16 (53)	0.36
Hyperlipidemia, n (%)	50 (36)	137	42 (39.3)	8 (26.7)	0.20
DM, n (%)	37 (27)	137	27 (25.2)	10 (33.3)	0.38
CKD, n (%)	48 (35)	137	32 (30)	16 (53)	0.02
CO, L/min (IQR)	4.1 (3.5–4.7)	73	4.1 (3.5–4.7)	3.4 (3.0–4.5)	0.30
PVR, WU (IQR)	2.3 (1.8–3.2)	73	2.3 (1.8–3.2)	2.5 (1.7–3.6)	0.89
Hospitalization time, days (IQR)	38 (26–61)	137	38 (26–65)	36 (26–58)	0.54
ICU hospitalization time (IQR)	7 (4–13)	137	7 (5–13)	6 (3–11)	0.08

IQR—interquartile range, BMI—body mass index, SD—standard deviation, HF—heart failure, DM—diabetes mellitus, CKD—chronic kidney disease, CO—cardiac output, PVR—pulmonary vascular resistance, ICU—intensive care unit.

**Table 2:** Characteristics of the deceased.

Parameter	HTx (*n* = 19)	LVAD (*n* = 9)
Age, years (IQR)	60 (35–66)	58 (51–65)
BMI, kg/m^2^ (SD)	26.5 (4.9)	29.5 (4.0)
Body mass, kg (SD)	76.9 (19.9)	93.5 (10.2)
Ischemic HF etiology, n (%)	11 (58)	6 (67)
Sex, male (%)	16 (84)	9 (100)
Hypertension, n (%)	8 (42)	7 (78)
Hyperlipidemia, n (%)	6 (32)	2 (22)
DM, n (%)	6 (32)	5 (56)
CKD, n (%)	5 (26)	4 (44)
CO, L/min (IQR)	4.25 (3.83–4.8)	4.12 (3.00–5.23)
PVR, WU (IQR)	2.4 (2.1–3.8)	2.3 (1.5–3.0)
Hospitalization time, days (IQR)	29 (17–71)	36 (26–58)
ICU hospitalization time (IQR)	12 (3–18)	3 (2–14)

IQR—interquartile range, BMI—body mass index, SD—standard deviation, HF—heart failure, DM—diabetes mellitus, CKD—chronic kidney disease, CO—cardiac output, PVR—pulmonary vascular resistance, ICU—intensive care unit.

**Table 3:** Average laboratory parameters—baseline.

Parameter	All Patients (*n* = 137)	Valid *n*	HTx (*n* = 107)	LVAD (*n* = 30)	*p*-Value
MELD XI (IQR)	11.7 (9.4–16.7)	137	11.7 (9.2–16.7)	13.0 (9.6–16.1)	0.52
Hgb, g/dL (IQR)	12.7 (10.9–14.2)	133	12.8 (10.6–14.3)	12.5 (11.4–14.1)	0.75
Plt, k/µL (IQR)	200.0 (163.2–250.7)	133	196.1 (154.5–250.7)	207.3 (180.0–258.5)	0.09
INR (IQR)	1.3 (1.1–1.7)	134	1.3 (1.1–1.7)	1.4 (1.2–1.8)	0.58
APTT, s (IQR)	36.9 (32.3–45.4)	132	37.9 (33.0–45.9)	34.5 (30.7–41.6)	0.10
D-Dimer, mg/L (IQR)	2.1 (1.1–5.7)	117	2.1 (1.1–5.8)	2.1 (0.7–4.6)	0.64
Bilirubin, mg/dL (IQR)	1.3 (0.8–2.0)	131	1.3 (0.8–2.0)	1.1 (0.8–2.1)	0.89
ALAT, IU/L (IQR)	29.0 (21.3–51.0)	131	29.0 (21.1–51.6)	35.4 (22.5–50.0	0.59
Aspat, IU/L (IQR)	39.3 (25.3–59.0)	132	39.7 (25.0–61.0)	38.6 (25.5–58.2)	0.93
ALP, IU/L (IQR)	89.0 (62.5–108.5)	124	89.0 (62.5–105.6)	91.3 (66.3–121.3)	0.49
CK, IU/L (IQR)	159.0 (54.5–461.0)	115	198.4 (57.8–476.3)	119.0 (54.0–312.0)	0.52
Glucose, mg/dL (IQR)	103.6 (94.4–121.4)	124	105.7 (94.8–121.3)	98.8 (91.0–122.0)	0.30
Creatinine, mg/dL (IQR)	1.2 (1.0–1.4)	133	1.1 (0.9–1.4)	1.2 (1.0–1.5)	0.23
Uric acid, mg/dL (IQR)	6.4 (5.3–8.4)	112	6.2 (5.3–8.3)	6.4 (5.6–8.7)	0.40
Total protein, g/dL (SD)	6.6 (5.9–7.3)	88	6.8 (5.9–7.4)	6.3 (5.9–6.9)	0.18
Albumin, g/dL (IQR)	3.5 (3.0–3.9)	119	3.5 (2.9–4.0)	3.5(3.1–3.9)	0.94
CRP, mg/L (IQR)	13.4 (3.8–48.2)	132	13.7 (3.5–55.5)	13.1 (5.8–39.2)	0.64
LDL, mg/dL (IQR)	63 (47.5–78)	59	66 (46–75)	62 (50–86)	0.80
TG, mg/dL (IQR)	100.0 (83.0–130.0)	65	102.8 (84.5–132.5)	99.0 (82.0–107.0)	0.24
Na, mmol/L (IQR)	136.3 (133.7–139.7)	69	138.6 (136.4–139.9)	138.1 (135.3–139.9)	0.75
K, mmol/L (IQR)	4.1 (3.8–4.4)	69	4.3 (4.1–4.5)	4.2 (3.9–4.6)	0.30
PCT, ng/L (IQR)	0.13 (0.05–0.41)	130	0.12 (0.04–0.50)	0.15 (0.08–0.20)	0.97
Troponin, ng/L (IQR)	36 (15–959)	126	34 (13–1106)	39 (18–190)	0.95
NTproBNP, pg/mL (IQR)	4665 (2270–8975)	119	4269 (2086–8424)	7938.(3514–16617)	0.02

MELD—model for end stage liver disease, Hgb—hemoglobin, Plt—platelets, INR—international normalized ratio, APTT—activated partial thromboplastin clotting time, ALAT—alanine aminotransferase, Aspat—aspartate aminotransferase, ALP—Alkaline Phosphatase, CK—creatine kinas-, CRP—C-reactive protein, LDL—low-density lipoprotein, TG—triglycerides, PCT—procalcitonin, NTproBNP—N-terminal pro B-type natriuretic peptide, IQR—interquartile range, SD—standard deviation.

**Table 4:** Pharmacotherapy before HTx.

Pharmacotherapy	Valid *n*	Before the HTx
ACEI, n (%)	103	41 (40)
Beta-blocker, n (%)	103	85 (83)
Ivabradine, n (%)	103	11 (11)
ARNI, n (%)	103	26 (25)
MRA, n (%)	103	72 (70)
Digoxin, n (%)	103	10 (10)
Calcium antagonist, n (%)	103	8 (8)
Amiodarone, n (%)	103	8 (8)
ASA, n (%)	103	19 (18)
Statin, n (%)	103	60 (58)
Metformin, n (%)	103	15 (15)
SGLT2-inhibitor, n (%)	103	51 (50)
Anticoagulation, n (%)	103	68 (66)
VKA, n (%)	103	13 (13)
IPP, n (%)	103	62 (60)
Loop diuretic, n (%)	103	79 (77)
Thiazide diuretic, n (%)	100	11 (11)

**Table 5:** Pharmacotherapy before LVAD implantation.

Pharmacotherapy	Valid *n*	Before the LVAD
ACEI, n (%)	30	8 (27)
Beta-blocker, n (%)	30	23 (77)
Ivabradine, n (%)	30	3 (10)
ARNI, n (%)	30	8 (27)
MRA, n (%)	29	21 (72)
Digoxin, n (%)	30	1 (3)
Calcium antagonist, n (%)	30	9 (30)
Amiodarone, n (%)	30	3 (10)
ASA, n (%)	30	9 (30)
Statin, n (%)	30	18 (60)
Metformin, n (%)	30	4 (13)
SGLT2-inhibitor, n (%)	30	23 (77)
Anticoagulation, n (%)	30	13 (43)
VKA, n (%)	30	6 (20)
IPP, n (%)	30	23 (77)
Loop diuretic, n (%)	30	25 (83)
Thiazide diuretic, n (%)	30	8 (27)

**Table 6:** MELD-XI differences between HTx and LVAD groups—baseline and 1, 3, 6, and 12 months after.

MELD-XI	HTx	LVAD	*p*-Value
baseline	11.7 (9.2–16.7)	13.0 (9.6–16.1)	0.50
1 month post	14.8 (9.4–17.7)	11.2 (7.3–14.9)	0.02
3 months post	9.3 (6.7–13.3)	9.4 (6.4–13.6)	0.90
6 months post	9.4 (6.7–12.5)	12.0 (6.7–17.0)	0.20
12 months post	10.4 (7.2–12.9)	12.2 (6.5–14.1)	0.80

**Table 7:** Differences in MELD in the LVAD and HTx groups compared to the values at the baseline.

MELD-XI	Baseline	3 Months Post	6 Months Post	12 Months Post	*p*-Value	Test Statistic
LVAD	13.0 (9.6–16.1)	9.4 (6.4–13.6)	12.0 (6.7–17.0)	12.2 (6.5–14.1)	0.00	37.6
HTx	11.7 (9.2–16-7)	9.3 (6.7–13.3)	9.4 (6.7–12.5)	10.4 (7.2–12.9)	0.00	14.1
**MELD-3.0**						
HTx	12.7 (5.6–16.2)	5.6 (2.7–11.1)	3.3 (2.6–4.4)	5.3 (3.2–9.5)	0.00	24.2

**Table 8:** Laboratory parameters of liver and kidney function at the baseline and 3, 6, and 12 months after LVAD procedure.

LVAD	Baseline	3 Months Post [*p*-Value]	6 Months Post [*p*-Value]	12 Months Post [*p*-Value]
ALT	35.4 (22.5–50.0)	24.2 (19.6–29.5) [0.04]	20.3 (17.0–28.8) [0.05]	26.3 (19.3–28.6) [1.00]
AST	38.6 (25.5–58.2)	27.0 (24.4–36.5) [0.10]	24.7 (21.2–31.8) [0.05]	24.5 (22.4–36.4) [0.58]
GGTP	114.5 (54.0–162.5)	100.5 (68,7–137.7) [0.82]	75.7 (45.0–96.9) [0.21]	53.0 (29.0–98.0) [0.03]
Bil	1.1 (0.8–2.1)	0.8 (0.5–1.1) [0.00]	0.7 (0.5–1.4) [0.15]	0.9 (0.5–1.5) [0.27]
Urea	52.0 (40.5–62.8)	49.5 (36.2–59.7) [0.40]	44.3 (35.0–84.7) [1.00]	40.0 (34.6–49.8) [0.28]
Creatinine	1.2 (1.0–1.5)	1.1 (0.9–1.4) [0.07]	1.2 (1.0–1.5) [0.33]	1.1 (1.0–1.4) [0.58]

**Table 9:** Laboratory parameters of liver and kidney function at the baseline and 3, 6, and 12 months after LVAD procedure—deceased.

LVAD	Baseline	3 Months Post	6 Months Post	12 Months Post
MELD-XI	8.9 (5.1–11.6)	15.3 (12.4–19.9)	11 (8.4–13.8)	10.3 (7.1–15.6)
ALT	37.5 (24.0–57.0)	25.5 (20.8–30.0)	20.0 (16.0–24.8)	25.0 (16.0–29.0)
AST	36.0 (31.0–52.0)	23.3 (19.0–35.8)	26.0 (20.0–30.0)	37.0 (22.0–38.0)
GGTP	59.3 (48.5–184.5)	61 (42–71.5)	40.3 (24.3–51)	34.0 (27.0–56.5)
Bil	1.0 (0.7–1.7)	0.7 (0.5–1.1)	0.8 (0.5–0.9)	1.3 (0.8–1.5)
Creatinine	1.1 (1.1–1.4)	1.0 (0.8–1.7)	1.0 (0.9–1.3)	1.1 (0.8–1.5)

**Table 10:** Laboratory parameters of liver and kidney function at the baseline and 3, 6, and 12 months after HTx procedure.

HTx	Baseline	3 Months Post [*p*-Value]	6 Months Post [*p*-Value]	12 Months Post [*p*-Value]
ALT	29.0 (21.1–51.6)	43.6 (23.6–63.6) [0.00]	21.6 (15.6–30.9) [0.00]	25.0 (17.0–37.3) [0.00]
AST	39.7 (25.0–61.0)	27.0 (20.4–38.4) [0.00]	21.3 (17.5–27.1) [0.00]	26.3 (21.0–34.0) [0.00]
GGTP	81.6 (42.5–151.3)	141.6 (84.8–239.9) [0.73]	74.5 (43.5–159.0) [0.00]	49.0 (32.0–123.7) [0.00]
Bil	1.3 (0.8–2.0)	0.8 (0.6–1.3) [0.00]	0.6 (0.5–0.9) [0.00]	0.7 (0.5–0.9) [0.00]
Urea	50.5 (39.6–64.7)	64.0 (39.7–88.9) [0.60]	51.2 (39.3–64.7) [0.73]	51.3 (45.0–64.5) [0.29]
Creatinine	1.1 (0.9–1.4)	1.1 (0.9–1.7) [0.30]	1.2 (1.0–1.5) [0.50]	1.3 (1.1–1.6) [0.10]

**Table 11:** Laboratory parameters of liver and kidney function at the baseline and 3, 6, and 12 months after HTx procedure—deceased.

HTx	Baseline	3 Months Post	6 Months Post	12 Months Post
MELD-XI	12.4 (6.3–17)	7.4 (3.1–10.6)	10.7 (6.3–12.1)	15.2 (12.7–17.7)
ALT	29.0 (18.0–62.0)	20.5 (14.0–50.0)	23.5 (17.0–42.0)	31.3 (21.5–41.0)
AST	30.5 (25.0–74.0)	23.0 (15.5–26.5)	28.8 (24.3–32.5)	26.5 (20.0–33.0)
GGTP	101 (53–236)	194 (72–359)	104 (26–165)	76 (41–111)
Bil	1.2 (0.8–1.8)	0.8 (0.5–1.5)	1.0 (0.7–1.3)	1.0 (0.3–1.6)
Creatinine	1.2 (1.0–1.4)	1.3 (1.1–1.6)	1.2 (0.8–1.6)	2.5 (2.0–3.0)

**Table 12:** Complications

	HTx (%)	LVAD (%)	*p*-Value
3 months			
Mortality	17 (16)	8 (27)	0.14
Rehospitalizations	11 (15)	9 (53)	0.01
HF exacerbation	1 (1)	1 (6)	0.16
Infectious complications	34 (46)	6 (35)	0.38
6 months			
Mortality	17 (16)	9 (30)	0.07
Rehospitalizations	6 (9)	5 (33)	0.07
HF exacerbation	1 (1)	0 (0)	0.77
Infectious complications	11 (16)	4 (27)	0.06
12 months			
Mortality	19 (18)	9 (30)	0.11
Rehospitalizations	10 (15)	3 (33)	0.09
HF exacerbation	1 (1)	0 (0)	0.81
Infectious complications	10 (15)	4 (44)	0.05

## Data Availability

All generated data are included in the manuscript and Supplementary Materials.

## Supplementary Materials

The following supporting information can be downloaded at: https://www.mdpi.com/article/10.3390/jcm14175933/s1.

## Abbreviations

The following abbreviations are used in this manuscript:

ALT	alanine aminotransferase
AST	aspartate aminotransferase
BMI	body mass index
CKD	chronic kidney disease
CO	cardiac output
CRP	C-reactive protein
CVP	central venous pressure
HF	heart failure
HTx	heart transplantation
GFR	glomerular filtration rate
GGTP	gamma-glutamylotranspeptydase
INR	international normalized ratio
LVAD	left ventricular assist device
MPAP	mean pulmonary artery pressure
MELD	model of end-stage liver disease
MOF	multi-organ failure
NTproBNP	N-terminal pro B-type natriuretic peptide
PVR	pulmonary vascular resistance
VKA	vitamin K antagonists
