# Supplementary material for: Comparative Analysis of Multi-Organ Failure Trajectories Following Heart Transplantation and HeartMate 3 Implantation: A 1-Year Postprocedural Follow-Up Study Utilizing the MELD-XI Scale
Source: J Clin Med. 2025 Aug 22;14(17):5933. doi: 10.3390/jcm14175933 (PMC12428862; doi:10.3390/jcm14175933)

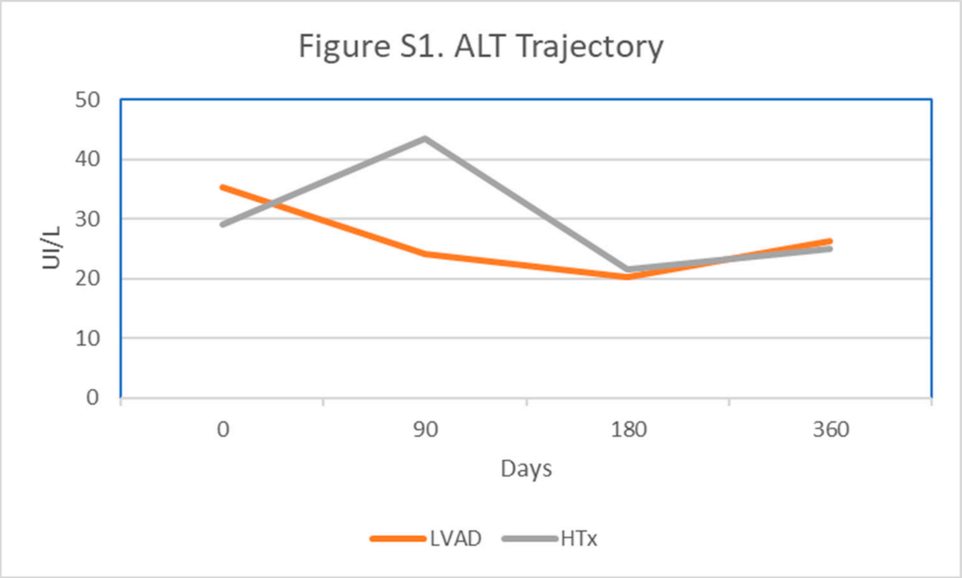

Figure S1. ALT Trajectory
UI/L
50
40
30
20
10
0
0
90
180
360
Days
LVAD
HTx

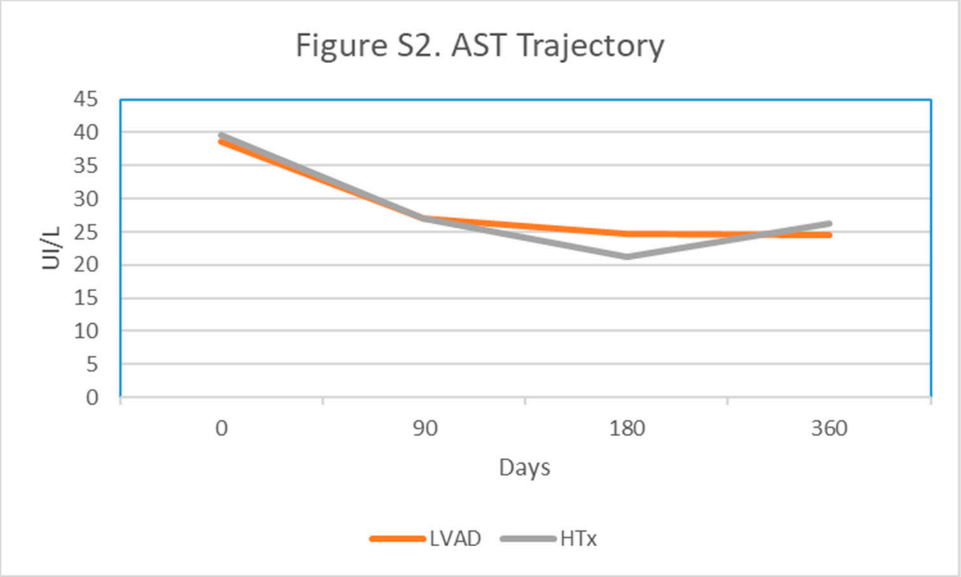

Figure S2. AST Trajectory
UI/L
45
40
35
30
25
20
15
10
5
0
0
90
180
360
Days
LVAD
HTx

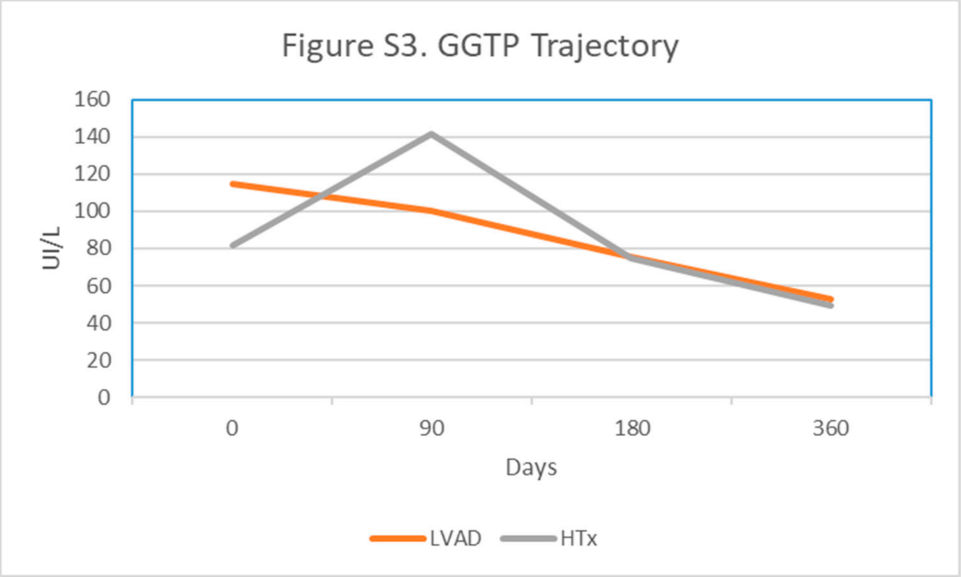

Figure S3. GGTP Trajectory
UI/L
160
140
120
100
80
60
40
20
0
0
90
180
360
Days
LVAD
HTx

Figure S4. Bilirubin Trajectory

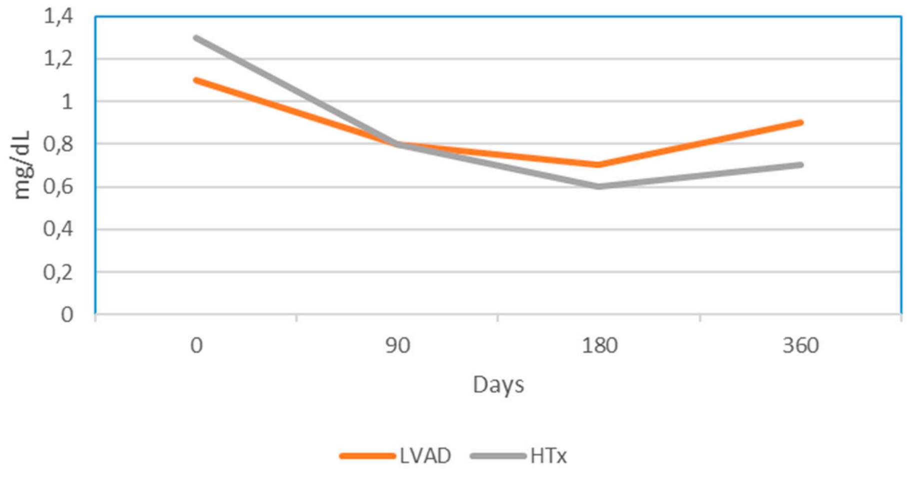

Figure S5. Urea Trajectory

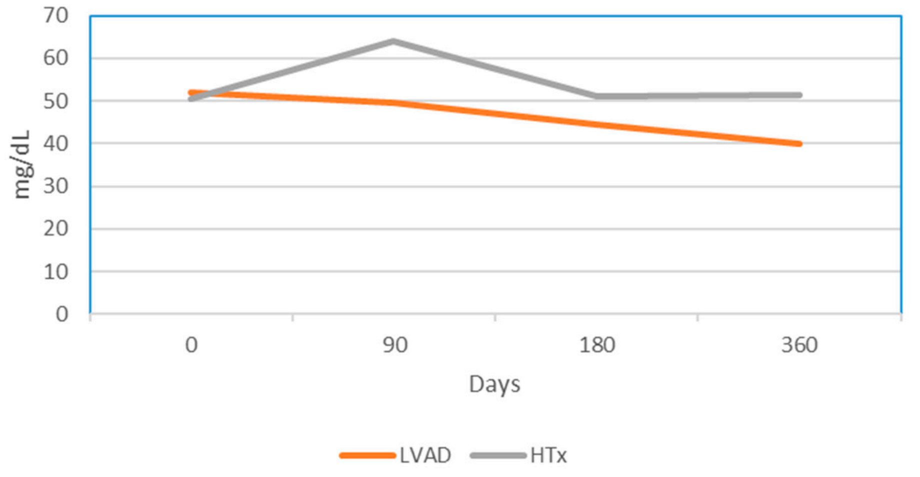

Figure S6. Creatinine Trajectory

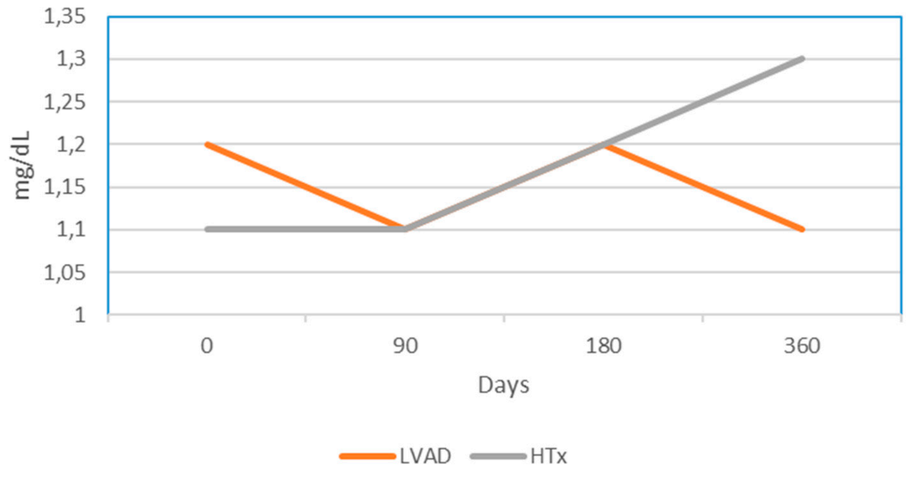

Supplement: Supplementary file 1 [file jcm-14-05933-s001.zip › jcm-3717593-supplementary.pdf]
